# Dysmenorrhœa and Sterility

**Published:** 1884-09

**Authors:** William Jefferson Guernsey

**Affiliations:** Philadelphia


### DYSMENORRHŒA AND STERILITY.—CURED WITH COLOCYNTHIS.

WM. JEFFERSON GUERNSEY, M. D., PHILADELPHIA.

Mrs. T. T., æt. twenty-six years, reported on the 17th of last January that, although her menstruation had been regular since her thirteenth year, she had never been "sick" without intense pain. The flow was somewhat scant and pale, and every twenty-four days. The pain did not precede the catamenia, but came *with* the flux. She had some headache and general malaise; also a too frequent micturition, especially at night, not infrequently being compelled to rise five or six times before morning. She had been a wife for two years and was much distressed by a fear that she would never become a mother. Her former physician (allopathic), after resorting to many experiments, had informed her that her only salvation was to "have the womb stretched." Before submitting to this piece of folly, however, she wisely yielded to the solicitation of a friend to try homœopathic treatment. She had but one *marked* symptom, which was, the great relief from pain by "doubling up" over any hard object. This, with an aggravation after eating, suggested Colocynthis. The 1M of Bœricke was given, with such happy results as to relieve the pain during her next menstruation and enable her to report an *absence* of any subsequent flow. She is now looking forward with much joy to the coming event that has already cast its shadow before.

